# Effect of Dapagliflozin and Magnesium Supplementation on Renal Magnesium Handling and Magnesium Homeostasis in Metabolic Syndrome

**DOI:** 10.3390/nu13114088

**Published:** 2021-11-15

**Authors:** Hwee-Yeong Ng, Wei-Hung Kuo, You-Lin Tain, Foong-Fah Leung, Wen-Chin Lee, Chien-Te Lee

**Affiliations:** 1 Division of Nephrology, Department of Internal Medicine, Kaohsiung Chang Gung Memorial Hospital, Chang Gung University College of Medicine, Kaohsiung 83301, Taiwan; kujiben@gmail.com (H.-Y.N.); b8701144@gmail.com (W.-H.K.); ffleong@cgmh.org.tw (F.-F.L.); leewenchin@gmail.com (W.-C.L.); 2 Department of Pediatrics, Kaohsiung Chang Gung Memorial Hospital, Chang Gung University College of Medicine, Kaohsiung 83301, Taiwan; tainyl@hotmail.com

**Keywords:** dapagliflozin, magnesium, TRPM6, kidney

## Abstract

The prevalence of metabolic syndrome (MetS) is increasing, and patients with MetS are at an increased risk of cardiovascular disease and diabetes. There is a close link between hypomagnesemia and MetS. Administration of sodium-glucose transporter 2 (SGLT2) inhibitors has been reported to increase serum magnesium levels in patients with diabetes. We investigated the alterations in renal magnesium handling in an animal model of MetS and analyzed the effects of SGLT2 inhibitors. Adult rats were fed a fructose-rich diet to induce MetS in the first 3 months and were then treated with either dapagliflozin or magnesium sulfate-containing drinking water for another 3 months. Fructose-fed animals had increased insulin resistance, hypomagnesemia, and decreased urinary magnesium excretion. Dapagliflozin treatment improved insulin resistance by decreasing glucose and insulin levels, increased serum magnesium levels, and reduced urinary magnesium excretion. Serum vitamin D and parathyroid hormone levels were decreased in fructose-fed animals, and the levels remained low despite dapagliflozin and magnesium supplementation. In the kidney, claudin-16, TRPM6/7, and FXDY expression was increased in fructose-fed animals. Dapagliflozin increased intracellular magnesium concentration, and this effect was inhibited by TRPM6 blockade and the EGFR antagonist. We concluded that high fructose intake combined with a low-magnesium diet induced MetS and hypomagnesemia. Both dapagliflozin and magnesium sulfate supplementation improved the features of MetS and increased serum magnesium levels. Expression levels of magnesium transporters such as claudin-16, TRPM6/7, and FXYD2 were increased in fructose-fed animals and in those administered dapagliflozin and magnesium sulfate. Dapagliflozin enhances TRPM6-mediated trans-epithelial magnesium transport in renal tubule cells.

## 1. Introduction

The prevalence and incidence of metabolic syndrome (MetS) are increasing, and MetS has been recognized as an important contributor to diabetes and cardiovascular morbidity [1]. The pathogenesis of MetS is multifaceted, and insulin resistance is a background factor that is linked to the presentation of MetS. Hyperinsulinemia also leads to various metabolic defects and disturbances [2].

Magnesium is involved in numerous important metabolic processes, including ATP-dependent biochemical reactions, synthesis of DNA, RNA expression, cell signaling at the muscle and nerve levels, and glucose and blood pressure control [3]. Serum magnesium levels are closely linked to glucose metabolism and insulin resistance. A reverse relationship was observed between magnesium intake and body mass index [4]. Several cohort studies have indicated a negative association between dietary magnesium intake and the risk of cardiovascular disease and total mortality [5]. A 0.1 mmol/L decrease in serum magnesium level was associated with an 18% increased risk of diabetes [6]. Low levels of serum magnesium have been observed in large cohorts of patients with type 2 diabetes [7], with a prevalence between 14% and 48%, compared to a prevalence ranging between 2.5% and 15% in healthy control subjects [8].

The kidney is the major organ that maintains magnesium homeostasis. Filtered magnesium is mainly reabsorbed in the loop of Henle and the distal nephron. In the thick ascending limb of the loop of Henle, claudin-16 and claudin-19 facilitate the passive reabsorption of both magnesium and calcium. In the distal nephron, TRPM6 and TRPM7 play an important role in the fine-tuning of magnesium regulation [9]. TRPM6 is highly regulated by epidermal growth factor (EGF). By binding to the EGF receptor, which is expressed at the basolateral membrane of distal tubule cells, the AKT/PI3K pathway is activated, resulting in the accumulation of TRPM6 at the apical membrane [10]. Several proteins have been identified as being involved in the extrusion of magnesium into the peritubular fluid at the basolateral membrane, including SLC41A1/2, CNNM2, and FXYD2 [11]. Recently, a new class of anti-diabetic therapeutics, sodium glucose transporter 2 (SGLT2) inhibitors, has demonstrated beneficial effects on diabetes [12]. A meta-analysis of 18 randomized trials, including four SGLT2 inhibitors, showed that these drugs can increase serum magnesium levels [13]. However, the effects of SGLT2 inhibitors on renal magnesium remain unclear.

In the present study, we aimed to analyze the alterations of magnesium transport in animals with MetS. A high fructose with low magnesium diet will be used to induce MetS. The effect of the SGLT2 inhibitor dapagliflozin on renal magnesium handling and magnesium homeostasis was investigated.

## 2. Materials and Methods

### 2.1. Animal Experiment

Adult male Sprague Dawley rats weighing 200–250 g were used in this experiment. The animals were maintained under a constant 12 h photoperiod at temperatures between 21 °C and 23 °C. The animals were allowed free access to water and food. The animals were allocated to control and fructose-diet groups. Fructose diet groups were fed a fructose-rich diet (60% fructose, 0.05% magnesium *wt/wt*, TD89427, Harlan Teklad, Madison, WI, USA), and control animals (*n* = 10) received standard rat chow for 6 months. In animals receiving a high-fructose diet, drug treatments were started after 3 months of feeding. The animals were divided into three groups as follows: 1. continued fructose feeding for six months (FR, *n* = 10); 2. continued fructose diet with dapaglifozin treatment (FR+Dapa, 1 mg·kg^−1^day^−1^ via oral gavage; Selleckchem, Houston, TX, USA, n = 10) for 3 months; 3. continued fructose diet with magnesium sulfate supplementation (FR+Mg, 296 mg/L of magnesium in drinking water, Taiwan Biotech Co., Ltd., Taoyuan, Taiwan, n = 10) for 3 months. Blood pressure of each animal was measured by indirect tail cuff method (Visitech BP2000, Apex, NC, USA) twice a week. At least 5 readings were obtained and averaged. Body weight was measured weekly. At the end of the study, 24 h urine samples were collected in an individualized metabolic cage. The rats were then sacrificed, and blood samples were withdrawn from the inferior vena cava for biochemical analysis. Animal protocols were approved by the Institutional Animal Care and Use Committee (IACUC), and all animal procedures were performed according to the International Committee on Animal Care and Use Committee (No. 2018050301).

### 2.2. Biochemical Assay

Levels of blood and urinary creatinine, electrolytes, uric acid, glucose, and insulin were measured using the SYNCHRON CX DELTA system (Beckman Coulter, Fullerton, CA, USA) according to the manufacturer’s protocol. Fractional excretion of magnesium was calculated as: (urine magnesium × plasma creatinine) divided by (plasma magnesium × urine creatinine) × 100. Serum vitamin D and intact parathyroid hormone (PTH) levels were determined using ELISA kits (CEA467Ge, USCN Life Science, Inc., Houston, TX, USA and 60–2500, Immutopics International, San Clemente, CA, USA, respectively).

### 2.3. Gene Expression Study

The kidney tissue was harvested for total RNA extraction using Direct-zol™ RNA Miniprep (Zymo Research, Irvine, CA, USA) following the manufacturer’s instructions, and spectrophotometry at a wavelength of 260 nm was used to detect total RNA concentrations. RNA was stored at −80 °C until use. A total of 0.1 μg of RNA from each sample was reverse-transcribed using a PrimeScript RT Reagent kit (Takara Biotechnology, Kusatsu, Shiga, Japan). Real-time PCR was performed using the Applied Biosystems™ 7500 Real-Time PCR Systems (Applied Biosystems, Forster, CA, USA) with Fast SYBR™ Green Master Mix (Applied Biosystems, V.A. Graiciuno, Vilnius, Lithuania) and primers for the target gene. The results of this study were normalized to the housekeeping gene, β-actin. The mRNA expression levels are presented as the ratio of each mRNA to β-actin mRNA. The primer set for the studied genes was as follows (Table 1):

### 2.4. Immunohistochemistry

Immunohistochemistry study was performed using the streptavidin-biotin complex method (TAHC02D kit, Biotna, Kaohsiung, Taiwan). Paraffin-embedded fixed tissue sections (4 μm) were deparaffinized with xylene and dehydrated with ethanol. Antigen retrieval was performed with 10 mM citrate buffer (pH 6.0) twice with 5 min of microwave treatment. The sections were incubated with 3% H_2_O_2_ in methanol for 10 min and then incubated with 10% serum for 30 min at room temperature. Then, anti-EGFR (1:1000, Abcam, Cambridge, UK), TRPM6 (1:800, Alomone Labs, Jerusalem, Israel), claudin-16 (1:500, Bioss Inc., Woburn, MA, USA), and FXYD2 (1:180, Proteintech Group, Inc., Rocky Hill, NJ, USA) antibodies were added, and the cells were incubated 4 °C overnight. The sections were incubated with the secondary antibody at room temperature for 30 min and then with peroxidase-conjugated streptavidin 37 °C for 30 min. Protein expression was visualized using 3,3′-diaminobenzidine tetrahydrochloride at a concentration of 30 mg/mL, containing 0.03% H_2_O_2_. All sections were photographed under a light microscope (×400) with a digital camera (DN100, E-600; Nikon, Tokyo, Japan). Immunostaining-positive areas were determined using Adobe Photoshop and quantified in 20 random fields per section using free ImageJ software (National Institutes of Health, Bethesda, MD, USA).

### 2.5. Cell Culture and Intracellular Magnesium Concentration Measurement

NRK52E cells (Bioresource Collection and Research Center, Hsinchu, Taiwan) were seeded on 96-well fluorescent plates, and cells were treated under the following conditions for 24 h: 1. regular medium; 2. 10 μM mesendogen (TRPM6 inhibitor; AOBIOUS Inc., Gloucester, MA, USA); 3. 10 μM AG1478 (EGFR inhibitor, Sigma-Aldrich, St. Louis, MO, USA); 4. 0.2 μM dapagliflozin (MedChemExpress, Monmouth Junction, NJ, USA); 5. 10 μM mesendogen and 0.2 μM dapagliflozin; 6. 10 μM AG1478 (0.2 μM) and dapagliflozin. The cells were then incubated with 5 μM Mg-Fura-2 AM (Thermo-Fisher Scientific, Waltham, MA, USA) at 37 °C for 60 min and then washed with the required final incubation medium three times. The cells were then incubated for a further 60 min to allow complete de-esterification of intracellular AM esters before fluorescence measurements. All experiments were repeated 4–6 times, and results were then averaged. The fluorescence was recorded at excitation/emission wavelengths of 330 nm/491 nm, and 369 nm/551 nm (Varioskan™ LUX multimode microplate reader, Thermo-Fisher Scientific). The Mg^2+^ concentration can be obtained using the following equation:

$$\left[Mg^{2+}\right]=K_{d}Q\frac{\left(R-R_{min}\right)}{\left(R_{max}-R\right)}$$

where R represents the fluorescence intensity ratio (F_λ1_/F_λ2_), where λ1 and λ2 are the fluorescence detection wavelengths for the ion-complexed and free indicators, respectively. R_min_ is the R value of zero magnesium, and R_max_ is the R value of magnesium saturation. Q is the ratio F_min_/F_max_ at λ2, and K_d_ is the dissociation constant for the ion-indicator complex.

### 2.6. Statistical Analysis

Data analysis was performed using SPSS Statistics version 17 (IBM, Armonk, NY, USA). Biochemical data are expressed as mean ± standard deviation (SD), and results of gene expression analysis and immunohistochemistry are shown as mean ± SEM. Comparisons between two groups were performed using Student’s *t*-test. Statistical significance was set at *p* < 0.05.

## 3. Results

### 3.1. Laboratory Data

Table 2 displays the laboratory data of the study animals. Animals fed a fructose diet for six months had significantly higher fasting glucose, insulin, HOMA-insulin resistance (IR), and triglyceride levels than the control group (*p* < 0.05). Serum magnesium levels were lower than those in the control group (*p* < 0.05). In animals treated with dapagliflozin and magnesium supplementation, lower glucose, insulin, and HOMA-IR levels were observed compared to those in the fructose group. Both treatment groups also had increased serum magnesium levels (both *p* < 0.05). The FEMg was significantly lower in fructose group than control animals (*p* < 0.05). Dapagliflozin treatment was associated with reduced FEMg than fructose group, and magnesium supplementation increased FEMg. Daily urinary magnesium excretion was significantly lower in the fructose group than in the control group (*p* < 0.05), and treatment with dapagliflozin reduced urinary magnesium excretion (*p* < 0.05). In contrast, magnesium supplementation increased urinary magnesium excretion. Animals treated with dapagliflozin also presented with lower body weight, lower blood pressure, and a higher daily urine excretion than fructose-fed animals (all *p* < 0.05). Magnesium supplementation in fructose-fed animals did not affect body weight, blood pressure, or urine amount relative to the fructose-only group. Serum intact parathyroid hormone (PTH) levels were significantly lower in the fructose group than in the control group (*p* < 0.05). Administration of dapagliflozin or magnesium supplementation did not reverse this decrease. A significant decrease in 1,25-dihydroxyvitamin D3 was noted in the fructose group compared to that in the control group (*p* < 0.05). Animals treated with dapagliflozin or magnesium supplementation were also associated with reduced 1.25-dihydroxyvitamin D3 levels (both *p* < 0.05, vs. control group).

### 3.2. Gene Expression Analysis

**Figure 1:**
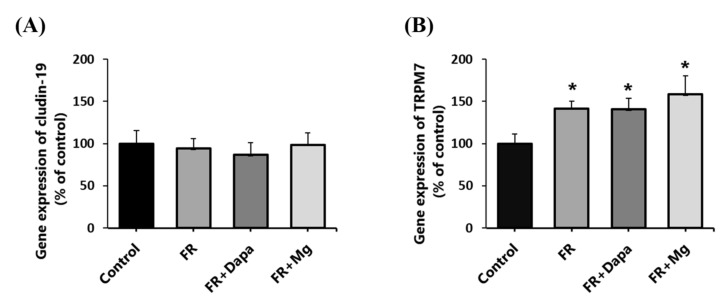
Gene expression analysis of claudin-19 (**A**) and TRPM7 (**B**) in different groups. FR: fructose group; FR+Dapa: fructose and dapagliflozin; FR+Mg: fructose and magnesium sulphate, * *p* < 0.05 vs. control.

Gene expression levels of claudin-19 (*CLDN19*) and *TRPM7* are depicted in Figure 1. Compared with the control group, claudin-19 expression did not change significantly (94.2 ± 11.8% of control). Treatment with dapagliflozin or magnesium sulfate supplementation did not affect its expression (FR+Dapa: 86.6 ± 14.7%; FR+Mg: 98.7 ± 14.1%, both *p* > 0.05). Increased *TRPM7* expression was noted in fructose-fed animals (141.7% ± 8.9% of control, *p* < 0.05). Dapaglilozin treatment and magnesium sulfate supplementation both did not prevent the increase in *TRPM7* (FR+Dapa: 140.6 ± 13.4%; FR+Mg: 158.4 ± 21.7% of control, both *p* < 0.05). There were no significant differences among the three groups.

### 3.3. Immunohistochemistry

**Figure 2:**
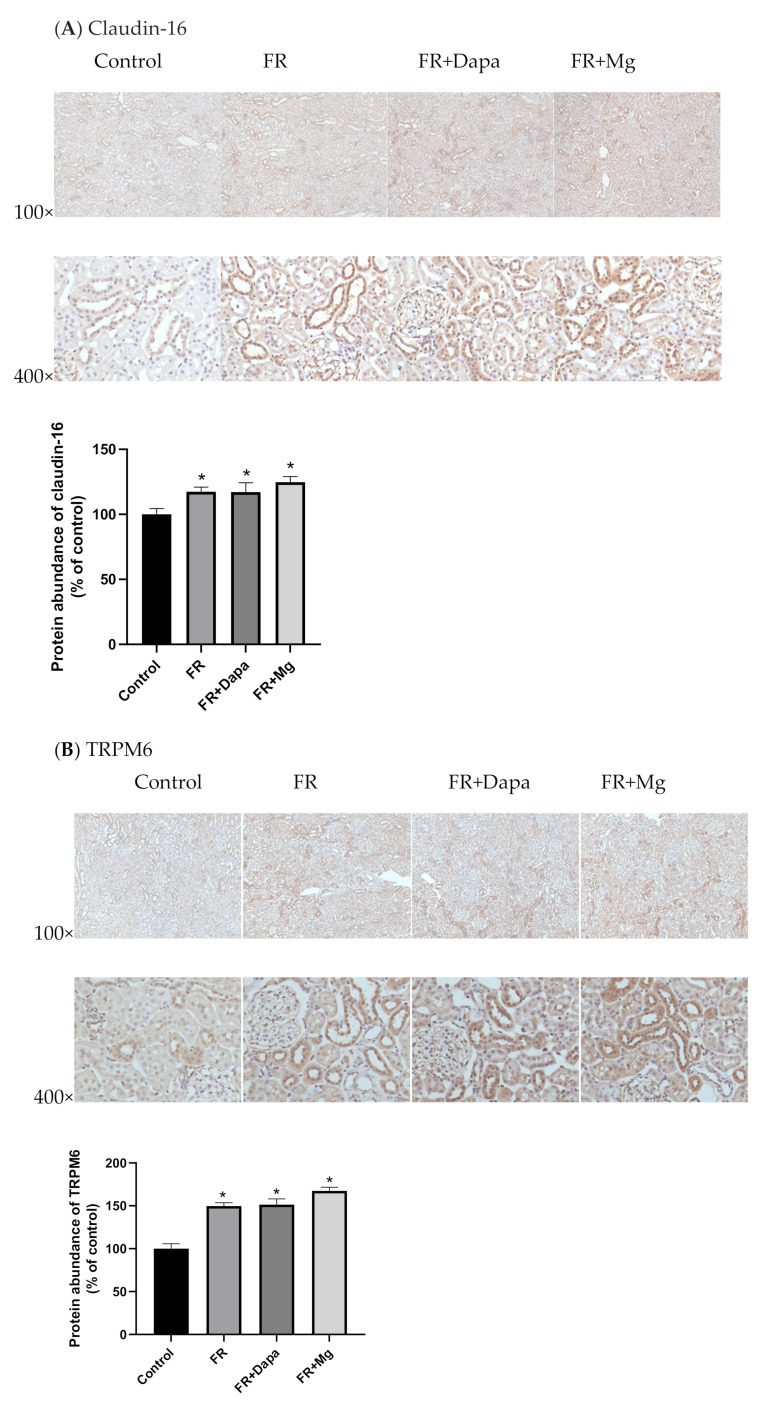

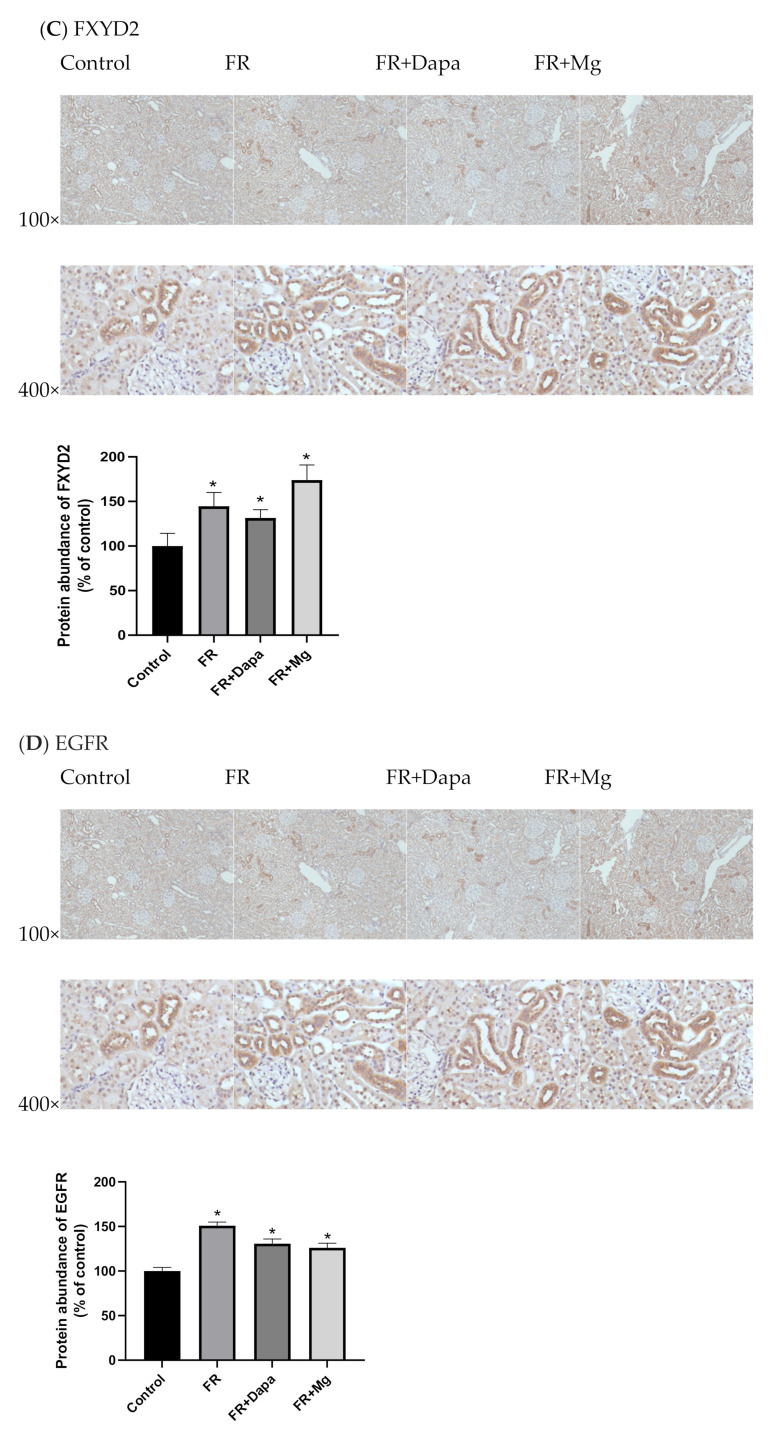
Protein abundance of claudin-16 (**A**), TRPM6 (**B**), FXYD2 (**C**), and EGFR (**D**) in renal tissue. FR: fructose group; FR+ Dapa: fructose and dapagliflozin; FR+Mg: fructose and magnesium sulphate, * *p* < 0.05 vs. control.

The protein abundance of claudin-16 was increased in the fructose group (112.4 ± 4.4% of control, *p* < 0.05, Figure 2A). Treatment of dapagliflozin or magnesium sulfate supplementation both did not prevent increase in claudin-16 (FR+Dapa: 127.3 ± 7.0%; FR+Mg: 124.8 ± 4.2%, *p* < 0.05 vs. control group). There were no significant differences among the three groups. Compared with control animals, the abundance of TRPM6 was increased in the fructose group (140.7 ± 3.9% of control, *p* < 0.05, Figure 2B). The increase was also noted in the dapagliflozin treatment (FR+Dapa: 151.4% ± 6.8%) and FR+Mg groups (167.4% ± 4.2%). The abundance of FXYD2 was increased in the FR group (144.5% ± 15.6% of control, *p* < 0.05, Figure 2C). Administration of dapagliflozin or magnesium sulfate-containing water was accompanied by increased FXYD2 (131.4% ± 11.4% and 173.9% ± 17.0% of the control group, both *p* < 0.05). A significant increase in EGFR was observed in the FR group (150.9% ± 4.0% of the control group, Figure 2D). Treatment with either dapagliflozin or magnesium supplementation did not reverse the increase in EGFR expression (FR+Dapa: 130.8 ± 5.1%; FR+Mg: 126.1 ± 5.2% of the control group, both *p* < 0.05).

### 3.4. Intracellular Magnesium Concentration

**Figure 3:**
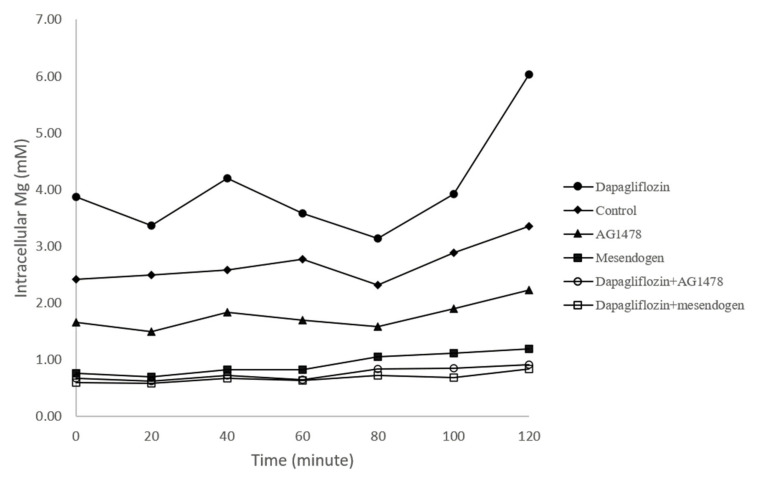
Measurement of intracellular magnesium (Mg) concentration.

After incubation for 24 h, the intracellular magnesium concentration was measured and calculated. As shown in Table 3 and Figure 3, treatment with dapagliflozin was associated with a 60% increase in intracellular magnesium concentration compared to the control group at the time of the first measurement (0 min). Treatment with both AG1478 and mesendogen alone significantly decreased the intracellular magnesium concentration. Combined dapagliflizin with either AG1478 or mesendogen both resulted in lower magnesium concentrations (both *p* < 0.05). Over 120 min, magnesium concentration was higher in the dapagliflozin group than in the control group, except at 80 min. AG1478 treatment alone was associated with significantly lower concentrations at 20, 60, 100, and 120 min. Mesendogen treatment alone decreased magnesium concentration throughout the 120 min period, except at 80 min. The combined treatment (dapagliflozin with AG1478 and dapagliflozin with mesendogen) significantly decreased the magnesium concentration at most time points.

## 4. Discussion

This study demonstrated that a high-fructose diet induced manifestations mimicking MetS. Low serum magnesium levels and reduced urinary magnesium excretion were observed in these animals. Administration of dapagliflozin or magnesium sulfate-containing water improved insulin resistance. Both interventions increased serum magnesium levels, and dapagliflozin further decreased urinary magnesium excretion. In the kidney, fructose feeding was associated with increased expression of claudin-16 and TRPM6/7. An in vitro study indicated that dapagliflozin enhanced renal epithelial magnesium transport through TRPM6.

Magnesium plays an important role in glucose metabolism, including insulin resistance and insulin secretion. In pancreatic β-cells, magnesium directly influences insulin secretion by regulating glucokinase activity [14]. Hypomagnesemia is associated with decreased insulin receptor activity by reducing the affinity of ATP to the insulin receptor, leading to increased insulin resistance [15]. Hypomagnesemia also increases inflammation and oxidative stress, which are common pathways that induce insulin resistance and MetS [16]. A previous study suggested that a fructose-combined magnesium deficiency diet induced MetS with amplified insulin resistance, inflammation, and oxidative stress [17]. In the present study, magnesium supplementation with magnesium sulfate-containing drinking water partially corrected the elevated HOMA-IR and triglyceride levels. Serum magnesium levels were also increased, with comparable urinary magnesium excretion. Dapagliflozin treatment reversed the features of MetS, including body weight and blood pressure. Contrary to magnesium supplementation, decreased urinary magnesium excretion was noted, indicating enhanced magnesium reabsorption during dapagliflozin administration. This beneficial effect may not only reverse hypomagnesemia-associated deleterious effects [16]; it may also contribute to prevention of arrhythmia in patients with heart failure and improve long-term outcome [18].

Renal handling contributes to the regulation of magnesium homeostasis. Our understanding of the molecular transport mechanism of magnesium has advanced significantly in recent decades [19]. Claudins are intercellular transporters through which paracellular transport occurs [20]. Increased claudin-16 expression was observed in fructose-fed animals and in the other two interventional groups, indicating increased paracellular transport of magnesium in Henle’s loop. Major magnesium transporters expressed in distal segments, such as TRPM6/7 and FXYD2, were increased in the fructose group as well as in the dapagliflozin and magnesium sulfate groups. The increase in fructose group represents renal adaptation in response to magnesium deficiency [21]. Recently, the beneficial effect of SGLT2 inhibitors in correcting hypomagnesemia has drawn the interest of researchers. Patients with lower serum magnesium levels at baseline were unexpectedly associated with an increase in magnesium levels following treatment with SGLT2 inhibitors [22]. A number of possible mechanisms have been proposed, including increased renal magnesium absorption due to increased glucagon levels, improvement of insulin resistance, and shifting of intracellular magnesium to the extracellular volume due to reduced insulin levels and increased aldosterone levels [23,24]. Although a regulating mechanism of TRPM6 by insulin or glucagon has been proposed, the direct evidence is lacking [25]. Our results showed that dapagliflozin upregulated renal magnesium transporters, including claudin-16 and TRPM6/7, resulting in decreased magnesium excretion. It is therefore indicated that both paracellular and transcellular transport were increased [26]. Moreover, the in vitro study by monitoring intracellular magnesium concentration suggested dapagliflozin increased trans-epithelial magnesium transport via TRPM6. This finding was also supported by the inhibitory effects of antagonists of EGFR and TRPM6. Nevertheless, further study is required to elucidate its molecular mechanism.

Excessive fructose intake can inhibit 1,25(OH)2 vitamin D3 synthesis. The direct mechanism of fructose-induced decrease in 1,25(OH)_2_D3 levels is due to a decrease in CYP27B1 (1-alpha-hydroxylase) expression [27]. It has been reported that vitamin D3 can stimulate intestinal magnesium absorption [28]. Furthermore, synthesis of vitamin D by hepatic 25-hydroxylation and renal 1-alpha hydroxylation is a magnesium-dependent process [29]. However, our results revealed that vitamin D3 levels remained low despite increased magnesium levels due to either dapagliflozin or magnesium supplementation. This finding suggests that fructose feeding is the major factor that suppresses vitamin D synthesis. The relationship between PTH and magnesium levels is complex. Magnesium can bind to calcium-sensing receptors and inhibit its signaling in the parathyroid gland, resulting in a decrease in PTH synthesis and secretion [30]. Profound magnesium depletion suppresses the release of PTH and induces skeletal resistance to PTH [31]. In the kidney, PTH increases renal magnesium absorption [32]. A significant decrease in PTH was noted in fructose-fed animals and in the other two interventional groups. It appears that the decreased PTH level in our experiment was not related to the alteration of magnesium. One recent clinical study highlighted the high prevalence of vitamin D deficiency in MetS, and a blunted parathyroid response was suspected [33]. Further study is mandatory to evaluate the systemic influence of decreased vitamin D and parathyroid hormone if dapagliglozin modulates intestinal magnesium resorption by TRPM6 transport.

## 5. Conclusions

High fructose combined with low magnesium diet induced MetS with associated hypomagnesemia. Increased expression of renal magnesium transporters, including claudin-16, TRPM6/7, and FXYD2, was noted in the experimental animals. Treatment with dapagliflozin or magnesium sulfate supplementation improved insulin resistance and increased serum magnesium levels. Dapagliflozin reduced urinary magnesium excretion and enhanced trans-epithelial magnesium transport through TRPM6 in renal tubule cells.

## Figures and Tables

**Table 1:** The primer set for the studied genes.

Gene	Forward (5′-3′)	Reverse (5′-3′)
Claudin-*19*	TCATATCCAGTCAGCAAGA	AGCAGTCAAAGTACAGAGA
*TRPM7*	GTTCAGAGGACCATCAAGA	CAAGAGCATCAAGCATAGC

**Table 2:** Laboratory data of study animals.

	Control (*n* = 10)	FR (*n* = 10)	FR+Dapa (*n* = 10)	FR+Mg (*n* = 10)
Body weight (g)	586.6 ± 56.5	597.4 ± 57.2	521.6 ± 21.6 *^,#^	615.1 ±72.0
Blood pressure (mmHg)	149.4 ± 2.6	155.6 ± 7.7	145.5 ± 6.9 ^#^	155.7 ±6.2
Water intake (mL/day)	52.2 ± 11.0	42.5 ± 7.6	52.0 ± 3.6	40.1 ±12.4
24h Urine (mL/day)	29.4 ± 10.9 ^#^	20.0 ± 8.4 *	41.7 ± 12.7 *^,#^	17.9 ±7.6 *
Blood Glucose (mg/dL)	109.0 ± 14.3	134.6 ± 22.4 *	99.6 ± 12.5 ^#^	109.1 ± 10.2 ^#^
Blood Triglyceride (mg/dL)	70.7 ± 27.1	231.0 ± 137.9 *	131.0 ± 62.8 ^#^	192.1 ± 59.8 *
Blood Insulin (μU/mL)	3.0 ± 2.2	11.6 ± 9.2 *	4.2 ± 1.4 ^#^	7.3 ± 2.7 *^,#^
HOMA-IR	0.8 ± 0.6	3.9 ± 3.1 *	1.0 ± 0.3^#^	1.6 ± 0.6^#^
Blood Creatinine (mg/dL)	0.29 ± 0.05	0.26 ± 0.05	0.27 ± 0.04	0.30 ± 0.06
Blood Calcium (mg/dL)	9.1 ± 0.7	9.2 ± 0.7	9.2 ± 0.4	9.9 ± 0.5
Blood Mg (mg/dL)	2.4 ± 0.3	1.8 ± 0.2 *	2.1 ± 0.4 *^,#^	2.2 ± 0.2^#^
Blood i-PTH (pg/mL)	326.6 ± 104.1	149.9 ± 32.6 *	175.2 ± 50.0 *	181.9 ± 63.8 *
Blood 1,25-dihydroxyvitamin-D3 (pg/mL)	71.0 ± 19.8	53.6 ± 11.6 *	54.2 ± 20.5 *	56.8 ± 8.9 *
FEMg(%) Urine Mg (mg/24 h)	4.1 ± 2.9 19.6 ± 7.3	2.7 ± 1.4 * 10.1 ± 4.9 *	1.9 ± 1.3 *^,#^ 5.3 ± 3.6 *^,#^	6.7 ± 1.8 *^,#^ 19.6 ± 4.4 ^#^

FEMg: fractional excretion of magnesium; * *p* < 0.05 vs. control group; ^#^ *p* < 0.05 vs. FR group.

**Table 3:** Intracellular magnesium concentration (mM) under different conditions.

	0 min	20 min	40 min	60 min	80 min	100 min	120 min
Control	2.41 ± 0.68	2.5 ± 0.83	2.58 ± 1.05	2.77 ± 1.57	2.31 ± 0.77	2.88 ± 0.98	3.36 ± 0.69
Mesendogen	0.77 ± 0.41 *	0.71 ± 0.35 *	0.83 ± 0.43 *	0.83 ± 0.38	1.05 ± 0.52	1.12 ± 0.47 *	1.2 ± 0.62 *
AG1478	1.66 ± 0.71 *	1.49 ± 0.72 *	1.83 ± 1.04	1.69 ± 0.64	1.59 ± 0.55	1.90 ± 0.69 *	2.23 ± 0.86 *
Dapagliflozin	3.87 ± 1.85 *	3.36 ± 2.53	4.20 ± 2.74 *	3.58 ± 1.52 *	3.31 ± 0.62	3.92 ± 0.76 *	6.03 ± 1.84 *
Dapagliflozin+Mesendogen	0.60 ± 0.39 *	0.58 ± 0.38 *	0.67 ± 0.42 *	0.64 ± 0.34	0.73 ± 0.45	0.68 ± 0.36 *	0.84 ± 0.57 *
Dapagliflozin+AG1478	0.67 ±0.70 *	0.62 ± 0.65	0.73 ± 0.76 *	0.65 ± 0.77	0.84 ± 0.95 *	0.86 ± 1.01	0.91 ± 0.95 *

* *p* < 0.05 vs. control.

## Data Availability

The data presented in this study are available on request from the corresponding author.
