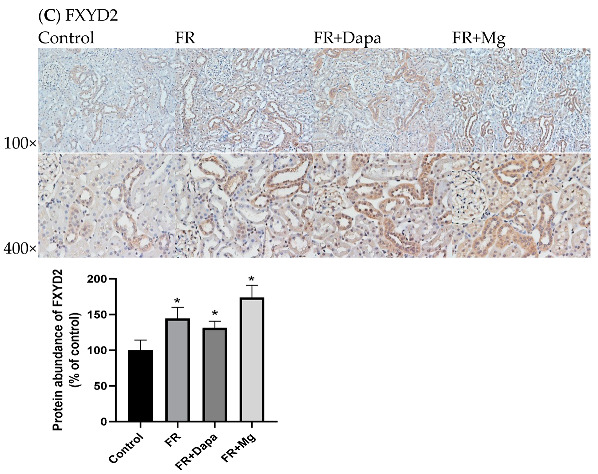# Correction: Ng et al. Effect of Dapagliflozin and Magnesium Supplementation on Renal Magnesium Handling and Magnesium Homeostasis in Metabolic Syndrome. *Nutrients* 2021, *13,* 4088

**DOI:** 10.3390/nu16193413

**Published:** 2024-10-09

**Authors:** Hwee-Yeong Ng, Wei-Hung Kuo, You-Lin Tain, Foong-Fah Leung, Wen-Chin Lee, Chien-Te Lee

**Affiliations:** 1 Division of Nephrology, Department of Internal Medicine, Kaohsiung Chang Gung Memorial Hospital, Chang Gung University College of Medicine, Kaohsiung 83301, Taiwan; kujiben@gmail.com (H.-Y.N.); b8701144@gmail.com (W.-H.K.); ffleong@cgmh.org.tw (F.-F.L.); leewenchin@gmail.com (W.-C.L.); 2 Department of Pediatrics, Kaohsiung Chang Gung Memorial Hospital, Chang Gung University College of Medicine, Kaohsiung 83301, Taiwan; tainyl@hotmail.com

## Error in Figure

In the original publication [1], there was a mistake in Figure 2C as published. During the preparation of the manuscript, images of the immunohistochemistry study were misplaced. The corrected Figure 2C appears below. The authors state that the scientific conclusions are unaffected. This correction was approved by the Academic Editor. The original publication has also been updated.